# The Prevention of Tubercles

**Published:** 1840-07

**Authors:** 


*On the Prevention of Tubercles.*—In a letter addressed to the Royal Academy of Medicine, M. Coster announces that, from certain experiments which he has made, he hopes to prove,

1. That it is possible, even in the face of predisposing causes, to prevent the development of the tubercular diathesis.
2. That even where the formation of tubercles has commenced, their progress may, in a great number of cases, be arrested.

The following are a few of the experiments upon which M. Coster has built up his hopes:

Two years ago he placed a number of dogs, rabbits, &c., in the circumstances most favorable to the development of the scrofulous diathesis. Thus, many of the unfortunate animals were shut up in dungeons, without light, incapable of moving, and exposed to a moist cold by means of wet sponges which were hung up in the cages. Some of the animals which were placed in these conditions, were fed on their ordinary diet; others were fed with ferruginous bread containing ½ oz. carbonate of iron to the pound. All the former became ill, the greater part tuberculous, but not one of those fed on the bread containing iron presented a trace of tubercles.—*London Lancet, from Bult. de l'Acad., Jan.* 31, 1840. *Med. Examiner, May* 16, 1840.

